# Increased phosphatidylcholine (16:0/16:0) in the folliculus lymphaticus of Warthin tumor

**DOI:** 10.1007/s00216-014-7890-9

**Published:** 2014-06-01

**Authors:** Qian He, Yoshinori Takizawa, Takahiro Hayasaka, Noritaka Masaki, Yukiko Kusama, Jiping Su, Hiroyuki Mineta, Mitsutoshi Setou

**Affiliations:** 1 Department of Otolaryngology-Head and Neck Surgery, First Affiliated Hospital of Guangxi Medical University, No. 6 Shuangyong Rd, Nanning, 530021 China; 2 Department of Otorhinolaryngology/Head & Neck Surgery, Hamamatsu University School of Medicine, 1-20-1 Handayama, Higashi-ku, Hamamatsu, Shizuoka 431-3192 Japan; 3 Department of Cell Biology and Anatomy, Hamamatsu University School of Medicine, 1-20-1 Handayama, Higashi-ku, Hamamatsu, Shizuoka 431-3192 Japan; 4 Department of Diagnostic Pathology, Hamamatsu University School of Medicine, 1-20-1 Handayama, Higashi-ku, Hamamatsu, Shizuoka 431-3192 Japan

**Keywords:** Warthin Tumor, Imaging mass spectrometry (IMS), Phosphatidylcholine, Palmitic acid

## Abstract

Warthin tumor (War-T), the second most common benign salivary gland tumor, consists mainly of neoplastic epithelium and lymphoid stroma. Some proteins and genes thought to be involved in War-T were evaluated by molecular biology and immunology. However, lipids as an important component of many tumor cells have not been well studied in War-T. To elucidate the molecular biology and pathogenesis of War-T, we investigated the visualized distribution of phosphatidylcholines (PCs) by imaging mass spectrometry (IMS). In our IMS analysis of a typical case, 10 signals were significantly different in intensity (*p* < 0.01) between the War-T and non-tumor (Non-T) regions. Five specific PCs were frequently found in the War-T regions of all of the samples: [PC (16:0/16:0) + K]^+^ (*m*/*z* 772.5), [PC (16:0/20:4) + K]^+^ (*m*/*z* 820.5), [PC (16:0/20:3) + K]^+^ (*m*/*z* 822.5), [PC (18:2/20:4) + K]^+^ (*m*/*z* 844.5), and [PC (18:0/20:5) + K]^+^ (*m*/*z* 846.5). PC (16:0/16:0) was increased specifically in the folliculus lymphaticus of War-T lymphoid stroma, suggesting a different metabolism. Localization of PC (16:0/16:0) might reflect inflammation activity participating in the pathogenesis of War-T. Thus, our IMS analysis revealed the profile of PCs specific to the War-T region. The molecules identified in our study provide important information for further studies of War-T pathogenesis.

## Introduction

Warthin tumor (War-T), also known as papillary cystadenoma lymphomatosum, is the second most common benign tumor of the salivary glands [1] and is usually found in males in their 60s or 70s. This tumor is found adjacent to the tail of the parotid gland and its periphery. These lesions account for 5–11 % of primary parotid neoplasms [2]. War-Ts are typically soft, undulant, and painless, showing slow growth. The rates of recurrence and malignant transformation are low [3]. Smoking is considered a strong risk factor for War-T, as most of War-T patients are smokers, and the duration and frequency of smoking are strongly related to the occurrence of War-T [4, 5].

War-Ts consist of two major structures: one is a bistratal neoplastic epithelium including basaloid and columnar oncocytic epithelial cells with cysts of various sizes, and the other is a lymphoid stromal component with infiltration of variable lymphocytes and lymph follicles. B lymphocytes and T lymphocytes are the main cell components of the lymphoid stroma of War-Ts [5, 6]. The tumor characteristics of War-T have been investigated in many studies. Although it has a polyclone origin, War-T is considered a neoplasm [7].

Many aspects of the molecular biology and pathogenesis of War-T are unclear, and various analyses have been performed to reveal the expression profile of proteins and genes in War-T, including those of cell adhesion molecules such as E-cadherin, HCAM, and ICAM-1 [8]. Regarding the lymphoid stroma of War-T, immunoglobulin heavy chain rearrangement and bcl-2 gene translocation were not found, indicating that the lymphoid stroma of War-T is benign and reactive [2]. Most of the molecular biology and pathogenesis investigations of War-T have focused on genomics and proteomics. Lipids are an important component of cells. About lipids of War-T, only a decreasing level of them in War-T comparing with normal tissue was revealed by Raman spectroscopy [9]. However there is no detailed information of lipids in War-T, and therefore, further analysis is required.

Lipids are involved in various physiological and pathological processes, and they provide significant information about metabolism. Phospholipids (PLs) are of particular interest as they are involved in the structure of biological membranes, proliferation, differentiation, metabolic regulation, and immunity [10]. Phosphatidylcholines (PCs), one type of PLs, are the major components of biological membranes, and they participate in many biological functions of cells. The importance of PCs and their fatty acyl residues has been described for many diseases, including tumors [11, 12], but not for War-T. Since the PCs containing different fatty acyl residues usually show distinct distributions [13], the distribution of each PC species in War-T should be determined. In addition, the elucidation of the profiles of PCs and their fatty acyl residues will provide important information about the molecular biology and pathogenesis of War-T (such as that concerning the neoplastic epithelium and lymphoid stroma) and increase our understanding of the character of War-T.

Pathology samples have been analyzed with imaging mass spectrometry (IMS). IMS is able to detect amounts of biomolecules and visualize their distributions in a single analysis on a tissue section without any labeling. Matrix-assisted laser desorption/ionization (MALDI)-time-of-flight (TOF) mass spectrometer is often used for the IMS analysis. MALDI is good at analyzing relatively large biomolecules such as lipids [13]. TOF can separate the difference even in the fatty acyl residues in PCs [14, 15]. Therefore, IMS based on MALDI-TOF is very useful for lipid analyses. This technique has successfully revealed that some specific phospholipids, especially PCs, were distributed in diseased regions and adjacent non-diseased regions in pathological tissue samples [15–17].

In the present study, we applied MALDI-TOF-IMS to find the specific PCs distributed in War-Ts consisting of neoplastic epithelium and lymphoid stroma and those in non-tumor (Non-T) regions. Two consecutive tissue sections were used for an IMS analysis and hematoxylin and eosin (HE) staining, respectively. The mass range in the IMS analysis was focused on PLs, and the peaks specifically detected in each region were examined by a statistical analysis. A variety of molecular distributions was shown visually by software, and the specific molecules were identified by tandem mass spectrometry (MS/MS).

## Materials and methods

### Clinical samples

Eight human samples (five from men and three from women) were analyzed. Two of the eight samples had both War-T and Non-T regions, and one sample had only a War-T region. Only a Non-T region was available and analyzed in the other five samples. The ages of the patients ranged from 30 to 78 years (Table 1). None of the patients had received medical treatment for War-T, and there was no recurrent patient. Samples were obtained in accord with protocols approved by the Hamamatsu University School of Medicine and diagnosed immediately after resection. The tissue samples were immediately frozen in liquid nitrogen-cooled isopentane without fixation and stored at −80 °C to maintain tissue morphology and minimize molecular degradation until their use in the IMS analysis.

**Table 1:** Patients’ information

Case no.	Age	Gender	Smoking information	Analyzed region
1	78	M	Smoker (but no detailed information)	War-T and Non-T
2	72	M	20 cigarettes/day for 30 years	War-T and Non-T
3	63	M	20 cigarettes/day for 20 years	War-T
4	57	M	–	Non-T
5	33	M	–	Non-T
6	61	F	–	Non-T
7	30	F	–	Non-T
8	40	F	–	Non-T

### Chemicals

Methanol, potassium acetate, ultrapure water, and carboxymethyl cellulose (CMC) sodium salt were purchased from Wako Chemicals (Osaka, Japan). 2,5-Dihydroxybenzoic acid (DHB) was purchased from Bruker Daltonics (Bremen, Germany). Standard peptides such as angiotensin II and bradykinin for the calibration of *m*/*z* values detected in the IMS analysis were purchased from Sigma-Aldrich (St Louis, MO, USA). All of the chemicals used were of the highest purity available.

### Imaging mass spectrometry

#### Tissue section preparation

The samples from cases 1, 4, and 7 were embedded in 2 % CMC at −80 °C before sectioning, and the other samples were sectioned without embedding. The tissue blocks were sliced to a thickness of 10 μm at −20 °C with a cryostat (CM1950; Leica, Wetzler, Germany). The consecutive tissue sections were mounted on an indium tin oxide (ITO)-coated glass slide (Bruker Daltonics) and a Matsunami Adhesive silane (MAS)-coated glass slide (Matsunami, Osaka, Japan) for the IMS analysis and HE staining, respectively. All slides with sample sections were stored at −20 °C until their use in the analyses.

#### Spray coating of the matrix solution

Matrix solution was prepared by dissolving 40 mg DHB in 1 mL of 70 % methanol with 20 mM potassium acetate. DHB matrix solution was sprayed uniformly by a 0.2-mm nozzle caliber airbrush (Procon Boy FWA Platinum; Mr. Hobby, Tokyo, Japan) maintaining a 15-cm distance from the tissue surface. Totally, 2 mL of DHB matrix solution was applied to each slide. DHB is a common matrix usually used to ionize lipids, such as PLs, by MALDI-IMS in the positive ion mode. Potassium salt is added to the matrix solution in order to form positive ions of [M + K]^+^ rather than [M + H]^+^ and [M + Na]^+^, and this can reduce the overlapping of signals from different molecules [18].

#### IMS conditions

We used a MALDI-TOF-TOF-type instrument (ultraflex II TOF/TOF, Bruker Daltonics) to analyze the tissue sections. An Nd:YAG laser (355-nm wavelength) was used for the desorption and ionization of PLs with a 200-Hz repetition rate. The setting parameters for the laser energy, detector gain, and random walk function were optimized manually for each measurement to obtain the highest sensitivity for the *m*/*z* range of 460–1,000. The calibration of *m*/*z* values detected in each IMS measurement was performed by using Mono DHB ([M + H]^+^, *m*/*z* 155.03), bradykinin ([M + H]^+^, *m*/*z* 757.40), and angiotensin II ([M + H]^+^, *m*/*z* 1046.54) in the positive ion mode. The measurement regions in the War-T and Non-T tissues were automatically scanned by the laser using flexControl (Bruker Daltonics). The number of laser shots at each measurement spot was 200, and the intervals between each measurement point were 200 μm. All data were acquired and visualized by flexImaging 2.1 software (Bruker Daltonics).

#### IMS data analysis

We converted the IMS dataset to the ANALYZE 7.5 format file in flexImaging and analyzed it using SIMtool software (in-house software; Shimadzu, Kyoto, Japan). The War-T and Non-T regions of interest (ROIs) were determined by comparison with the HE staining results of the consecutive tissue section. We chose the ROIs in the IMS data from case 1 as a representation, and the top 100 peaks were picked up within *m*/*z* 460–1,000 according to their intensities with the SIMtool software. After the exclusion of the matrix-derived peaks and isotopic peaks, 15 peaks were picked up in the mass range between *m*/*z* 740 and *m*/*z* 940 from the War-T and Non-T ROIs, respectively. The distributions of these peaks were visualized on a tissue section. The signal intensities of these peaks in each ROI were statistically compared by Welch *t* test. Differences with *p* < 0.01 were considered significant. These significant signals acquired from case 1 were reproduced independently from other cases, and the average among all the cases was also confirmed as significant. The experiments were repeated totally three times using consecutive sections.

### MS/MS analysis

We identified the characteristic peaks in the War-T and Non-T regions by MS/MS analyses performed on consecutive tissue sections in the positive ion mode by QSTAR Elite (Applied Biosystems/MDS Sciex, Foster City, CA). This instrument is a hybrid quadrupole-TOF mass spectrometer equipped with an orthogonal MALDI source with an Nd:YAG laser. The laser energy and collision energy were optimized to maximize the ionization of precursor ions and the characteristic fragmentation in MS/MS spectra. The PL species and its fatty acyl residues were determined by ion-fragment pattern referring to the candidate molecule hits in an MS Search on the Human Metabolome Database (http://www.hmdb.ca/spectra/spectra/ms/search).

## Results

### Histological observation of War-T specimens for case 1

**Fig. 1:**
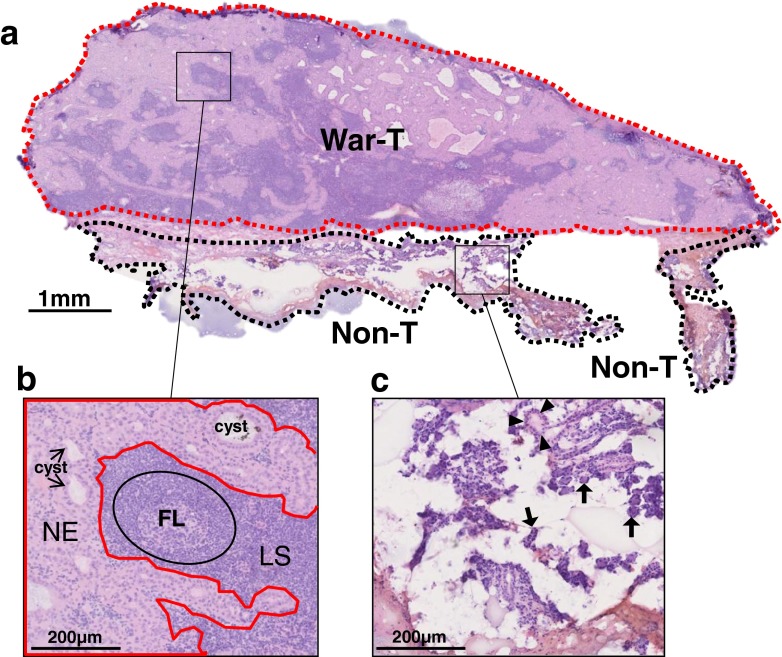
HE staining of a War-T tissue section of case 1. **a** Overall view of a frozen section. The upper part of the section (surrounded by the *red dotted line*) was occupied by War-T. The Non-T regions (indicated by *black dotted line*) were located in the lower part. **b** The enlarged image of the tumor region shows that the neoplastic epithelium (*NE*), lymphoid stroma (*LS*), and cysts are present. The neoplastic epithelium segregated by a *red line* appeared as light purple. Within the NE, cysts of various sizes were formed by epithelial cells. The LS containing numerous infiltrative lymphocytes separated by NE can be seen as a deep purple area in the War-T region, and it sometimes included folliculus lymphaticus (*FL*) (*black circle*). **c** Enlargement of a Non-T region shows many serous acini (*arrows*) and duct structures (*arrowheads*). *War-T*, Warthin tumor; *Non-T*, non-tumor

The samples were obtained from the parotid gland of the patients without any preoperative therapy. HE staining was performed to reveal the morphological characteristics of the War-T and Non-T regions. From the HE staining of case 1 shown in Fig. 1a, it is easy to see that the War-T region, which was occupied by neoplastic epithelium and lymphoid stroma, is on the upper side (surrounded by the red dotted line in the figure), and the Non-T region, which was characterized as normal epithelial cells and ductal structures, is on the bottom side (surrounded by black dotted line). The magnified image of the tumor side in Fig. 1a is shown as Fig. 1b, where the War-T region includes the neoplastic epithelium (NE) and lymphoid stroma (LS) separated by a red line. The NE surrounded by a red line showed a bistratified structure consisting of inside basaloid cells and outside columnar cells with abundant eosinophilic cytoplasm which was stained light purple, and this structure formed cysts of different sizes (shown as blank within the light purple region in Fig. 1b). The LS, containing infiltrative lymphocytes and folliculus lymphaticus (FL), was located outside of the red line boundary in Fig. 1b and was stained dark purple. Lymphocytes were recognized by the deeply stained nucleus and a relatively small amount of cytoplasm. FL (delineated by the black circle in Fig. 1b) with a paler germinal center and an outer corona was found in the LS.

Based on these features, we recognized lymphoid stroma in HE staining images. The Non-T region was distributed below the tumor region in Fig. 1a, and the enlarged image of this region is shown as Fig. 1c. From the HE staining, the Non-T region showed many serous acini, where glandular epithelial cells accumulate, and the duct structure appeared as a ring-like or streak-like structure. Each acinus (marked by an arrow in Fig. 1c) consisted of a single layer of glandular epithelial cells. The duct structures (marked by arrowheads in Fig. 1c) included striated ducts and interlobular ducts, which were formed by surrounding ductal epithelial cells. There were a few lymphocytes in the Non-T region.

According to the HE staining, the War-T and Non-T regions can be distinguished by their histological features. In addition, the neoplastic epithelium and lymphoid stroma can be distinguished in the War-T region. In the present study, all of War-T and Non-T regions were distinguished in this manner.

### Comparison of the mass spectra from the War-T and Non-T regions of case 1

**Fig. 2:**
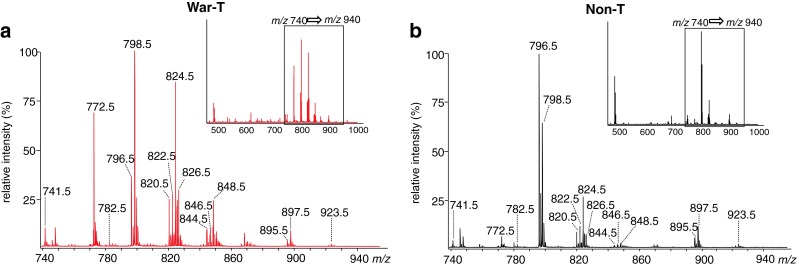
The mass spectra of War-T and Non-T from case 1. Each spectrum was averaged over War-T (**a**) and Non-T (**b**) regions distinguished in Fig. 1 and shown from *m*/*z* 740 to *m*/*z* 940 where signals from PLs are concentrated. The horizontal axis and the vertical axis indicate *m*/*z* and relative intensity, respectively. The *insets* in **a** and **b** show the overall mass spectra between *m*/*z* 460 and *m*/*z* 1000. Fifteen peaks marked by *labeled number* were detected from both regions. Each peak represents an individual molecule

The mass spectra from the War-T and Non-T regions of case 1 (Fig. 1) were averaged and are shown as Fig. 2a, b, respectively. The figure’s inset shows the average mass spectra obtained from the overall mass range, *m*/*z* 460–1,000. The top 100 peaks were picked up within this range by the SIMtool software. Matrix related and isotopic peaks were excluded. An enlargement of the mass range between *m*/*z* 740 and *m*/*z* 940 was from the region surrounded by the black line in overall mass spectrum, and the 15 representative peaks were labeled by *m*/*z* values. These peaks corresponding to PL species are commonly detected in the positive ion mode. From the entire mass spectra, a clear difference in signal intensity was observed in *m*/*z* 740–940 between the War-T and Non-T regions. In comparison with these mass spectra, most of the peaks were higher in War-T than Non-T, especially *m*/*z* 772.5 and *m*/*z* 824.5. In contrast, the peaks *m*/*z* 796.5 and *m*/*z* 897.5 were lower in War-T than Non-T.

### Ion images of typical signals from case 1

**Fig. 3:**
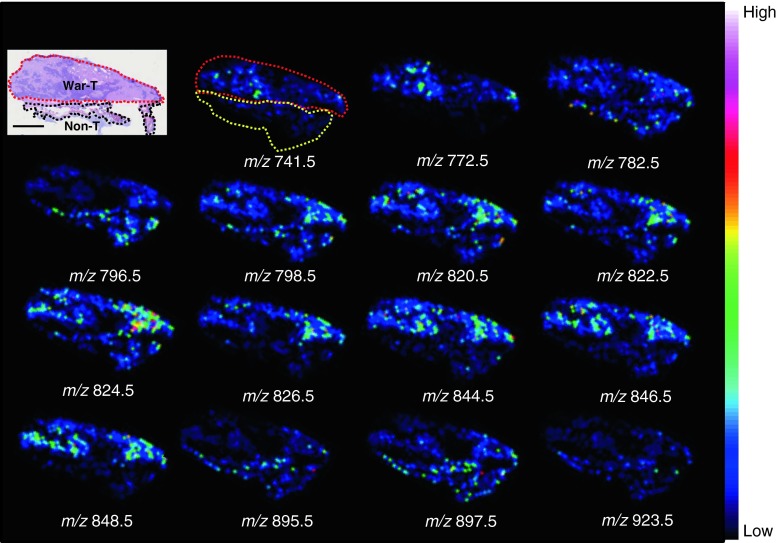
Visualization of molecular distributions for case 1. The ion images correspond to the 15 peaks which were detected between the War-T and Non-T regions. The distributions of these signals were nonuniform and showed some patterns in War-T and Non-T regions distinguished in the HE staining. Ion images corresponding to [PC (34:2) + K]^+^ (*m*/*z* 796.5), [TAG (52:3) + K]^+^ (*m*/*z* 895.5), [TAG (52:2) + K]^+^ (*m*/*z* 897.5), and [TAG (54:3) + K]^+^ (*m*/*z* 923.5) were located mainly in the Non-T region, whereas [PC (P34:1) + K]^+^ (*m*/*z* 782.5) and [PC (34:1) + K]^+^ (*m*/*z* 798.5) displayed no difference between the War-T and Non-T regions. The other nine signals were increased in the War-T region. *Scale bar* = 2 mm

To compare the spatial distribution of the 15 signals for the War-T and Non-T regions, we reconstructed ion images from these signals for case 1 (shown in Fig. 3). In a comparison with the average spectrum, ion images can make the location of each signal very clear on tissue sections. From the ion images, we found four signals (*m*/*z* 796.5, *m*/*z* 895.5, *m*/*z* 897.5, and *m*/*z* 923.5) located mainly in the Non-T region. The distributions of *m*/*z* 782.5 and *m*/*z* 798.5 appeared almost uniform. The other nine signals (*m*/*z* 741.5, *m*/*z* 772.5, *m*/*z* 820.5, *m*/*z* 822.5, *m*/*z* 824.5, *m*/*z* 826.5, *m*/*z* 844.5, *m*/*z* 846.5, and *m*/*z* 848.5) were accumulated in the War-T region.

According to the information from MS Search on the Human Metabolome Database, we speculated the assignments of these *m*/*z* values as follows: [SM (d34:1) + K]^+^ (*m*/*z* 741.5), [PC (32:0) + K]^+^ (*m*/*z* 772.5), [PC (P34:1) + K]^+^ (*m*/*z* 782.5), [PC (34:2) + K]^+^ (*m*/*z* 796.5), [PC (34:1) + K]^+^ (*m*/*z* 798.5), [PC (36:4) + K]^+^ (*m*/*z* 820.5), [PC (36:3) + K]^+^ (*m*/*z* 822.5), [PC (36:2) + K]^+^ (*m*/*z* 824.5), [PC (36:1) + K]^+^ (*m*/*z* 826.5), [PC (38:6) + K]^+^ (*m*/*z* 844.5), [PC (38:5) + K]^+^ (*m*/*z* 846.5), [PC (38:4) + K]^+^ (*m*/*z* 848.5), [TAG (52:3) + K]^+^ (*m*/*z* 895.5), [TAG (52:2) + K]^+^ (*m*/*z* 897.5), and [TAG (54:3) + K]^+^ (*m*/*z* 923.5).

### Statistical analysis of signal intensities from case 1

For the ROIs of case 1 analyzed by IMS, we compared the average intensities of these 15 detected peaks between the War-T and Non-T regions by using Welch’s *t* test. The average signal intensities, standard errors, and *p* values are shown for each *m*/*z* signal in Table 2. Ten of the 15 peaks showed a significant difference in signal intensity (*p* < 0.01) between the War-T and Non-T regions (highlighted as italic in Table 2). According to the results of the statistical analysis, the 10 signals were significantly different between War-T and Non-T. One signal, [PC (34:2) + K]^+^ (*m*/*z* 796.5), was higher in Non-T. The other nine signals, [SM (d34:1) + K]^+^ (*m*/*z* 741.5), [PC (32:0) + K]^+^ (*m*/*z* 772.5), [PC (36:4) + K]^+^ (*m*/*z* 820.5), [PC (36:3) + K]^+^ (*m*/*z* 822.5), [PC (36:2) + K]^+^ (*m*/*z* 824.5), [PC (36:1) + K]^+^ (*m*/*z* 826.5), [PC (38:6) + K]^+^ (*m*/*z* 844.5), [PC (38:5) + K]^+^ (*m*/*z* 846.5), and [PC (38:4) + K]^+^ (*m*/*z* 848.5), had higher signal intensity in War-T.

**Table 2:** Molecular screening in case 1, using Welch’s *t* test

*m*/*z*	Speculative assignment	Signal intensity		*p* value
		War-T	Non-T	
741.5	[SM (d34:1) + K]^+^	*8.0 ± 0.9*	3.2 ± 0.7	2.16 × 10^−5^
772.5	[PC (32:0) + K]^+^	*60.7 ± 6.3*	4.0 ± 0.8	9.85 × 10^−15^
782.5	[PC (P34:1) + K]^+^	2.2 ± 0.3	1.5 ± 0.3	0.09
796.5	[PC (34:2) + K]^+^	35.2 ± 3.0	*124.6 ± 18.4*	6.29 × 10^−5^
798.5	[PC (34:1) + K]^+^	96.1 ± 6.0	68.7 ± 11.3	0.04
820.5	[PC (36:4) + K]^+^	*23.9 ± 1.8*	7.6 ± 1.7	1.86 × 10^−9^
822.5	[PC (36:3) + K]^+^	*28.5 ± 2.4*	12.0 ± 2.1	1.68 × 10^−6^
824.5	[PC (36:2) + K]^+^	*83.1 ± 7.5*	33.8 ± 5.4	4.62 × 10^−7^
826.5	[PC (36:1) + K]^+^	*29.5 ± 2.7*	6.7 ± 1.5	1.83 × 10^−11^
844.5	[PC (38:6) + K]^+^	*9.1 ± 0.7*	1.5 ± 0.4	1.98 × 10^−15^
846.5	[PC (38:5) + K]^+^	*9.6 ± 0.8*	1.7 ± 0.4	2.50 × 10^−16^
848.5	[PC (38:4) + K]^+^	*21.5 ± 1.6*	2.3 ± 0.5	3.04 × 10^−21^
895.5	[TAG (52:3) + K]^+^	3.2 ± 0.4	6.1 ± 1.6	0.08
897.5	[TAG (52:2) + K]^+^	8.1 ± 0.8	12.6 ± 3.5	0.22
923.5	[TAG (54:3) + K]^+^	1.2 ± 0.2	1.6 ± 0.5	0.40

The signals shown in Figs. 2 and 3 were compared by Welch’s *t* test. Ten of the 15 molecules were significantly different in signal intensity (*p* < 0.01), set in italics. One signal, [PC (34:2) + K]^+^ (*m*/*z* 796.5), had higher signal intensity in Non-T, and all of the others had higher signal intensity in War-T

### Ion images of War-T signals in all cases

**Fig. 4:**
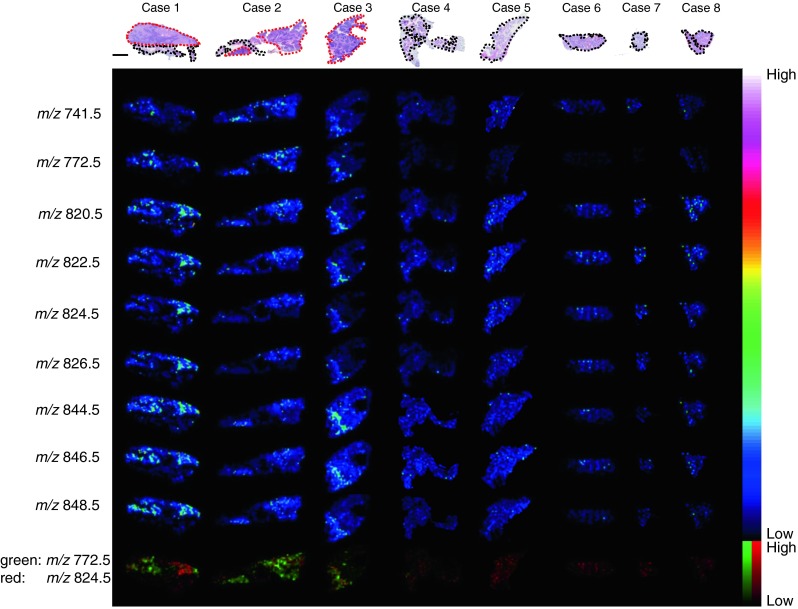
Ion images of War-T signals in all cases. The ion images for the nine War-T signals that were significantly different between the War-T and Non-T regions from case 1 were visualized in all cases. The HE staining images of each sample are shown on the *upper side*. The War-T and Non-T regions are indicated by a *red* and *black dotted lines*, respectively. From the ion images, an increase in signal intensities was found mainly in the War-T regions. Comparing the ion images with the HE staining images, the signals at [SM (d34:1) + K]^+^ (*m*/*z* 741.5) and [PC (32:0) + K]^+^ (*m*/*z* 772.5) were located mainly in the lymphoid stroma, and signals [PC (36:3) + K]^+^ (*m*/*z* 822.5), [PC (36:2) + K]^+^ (*m*/*z* 824.5), and [PC (36:1) + K]^+^ (*m*/*z* 826.5) were located in the neoplastic epithelium. Signals [PC (36:4) + K]^+^ (*m*/*z* 820.5), [PC (38:6) + K]^+^ (*m*/*z* 844.5), [PC (38:5) + K]^+^ (*m*/*z* 846.5), and [PC (38:4) + K]^+^ (*m*/*z* 848.5) seemed to be located in both regions. The merged image consisting of [PC (32:0) + K]^+^ (*m*/*z* 772.5) and [PC (36:2) + K]^+^ (*m*/*z* 824.5) revealed that these two signals came from different parts of the War-T region. *Scale bar* = 2 mm

These nine significant signals in War-T acquired from case 1 were used as candidates for specific signals selection. We applied these signals to all samples and visualized them as ion images to determine whether these signals were specific in all War-T (Fig. 4). According to these ion images, the signals [PC (36:3) + K]^+^ (*m*/*z* 822.5), [PC (36:2) + K]^+^ (*m*/*z* 824.5), and [PC (36:1) + K]^+^ (*m*/*z* 826.5) were located at the region which seemed to be neoplastic epithelium in the War-T region. The signals at [PC (36:4) + K]^+^ (*m*/*z* 820.5), [PC (38:6) + K]^+^ (*m*/*z* 844.5), [PC (38:5) + K]^+^ (*m*/*z* 846.5), and [PC (38:4) + K]^+^ (*m*/*z* 848.5) were located at a region which was considered to include neoplastic epithelium and lymphoid stroma. However, the signals at [SM (d34:1) + K]^+^ (*m*/*z* 741.5) and [PC (32:0) + K]^+^ (*m*/*z* 772.5) appeared partially in the War-T region and seemed to be involved in the lymphoid stroma structure. These signals were obscure in all Non-T regions.

Among the signals that were thought to be located in the neoplastic epithelium, the signal at [PC (36:2) + K]^+^ (*m*/*z* 824.5) showed the highest intensity in case 1 (Table 2). To clarify the different distributions of the signal at [PC (32:0) + K]^+^ (*m*/*z* 772.5), we merged the signals at [PC (32:0) + K]^+^ (*m*/*z* 772.5) and [PC (36:2)+K]^+^ (*m*/*z* 824.5). From the merged ion images, we found that the signals at [PC (32:0) + K]^+^ (*m*/*z* 772.5) and [PC (36:2) + K]^+^ (*m*/*z* 824.5) displayed different distributions, and this difference depended on the distinction of the structure in the War-T region. Finally, after we applied the War-T signals to all samples, those at [PC (32:0) + K]^+^ (*m*/*z* 772.5), [PC (36:4) + K]^+^ (*m*/*z* 820.5), [PC (36:3) + K]^+^ (*m*/*z* 822.5), [PC (38:6) + K]^+^ (*m*/*z* 844.5), and [PC (38:5) + K]^+^ (*m*/*z* 846.5) were found to be common to all War-T regions.

### Identification of molecules specifically detected in War-T regions

**Fig. 5:**
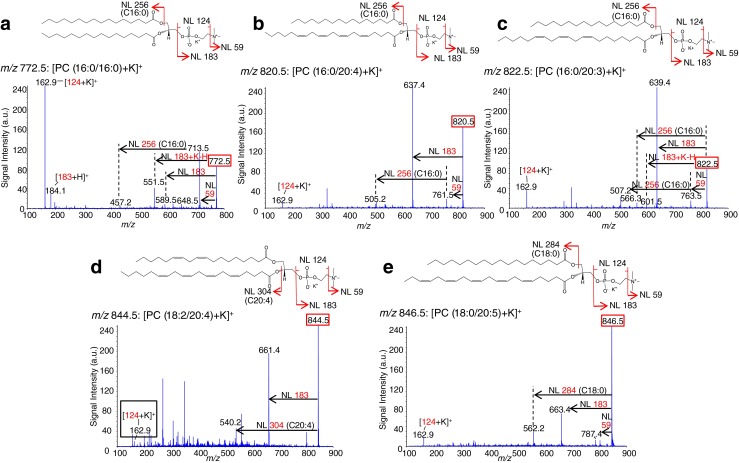
Molecular identification by the MS/MS analysis of tissue sections. The precursor ions were *m*/*z* 772.5 (**a**), *m*/*z* 820.5 (**b**), *m*/*z* 822.5 (**c**), *m*/*z* 844.5 (**d**), and *m*/*z* 846.5 (**e**). From the product ion spectrum, a molecule corresponding to *m*/*z* 772.5 was identified as [PC (16:0/16:0) + K]^+^. In the same manner, the signals at *m*/*z* 820.5, *m*/*z* 822.5, *m*/*z* 844.5, and *m*/*z* 846.5 were identified as [PC (16:0/20:4) + K]^+^, [PC (16:0/20:3) + K]^+^, [PC (18:2/20:4) + K]^+^, and [PC (18:0/20:5) + K]^+^, respectively

We then performed an MS/MS analysis to identify these five signals, [PC (32:0) + K]^+^ (*m*/*z* 772.5), [PC (36:4) + K]^+^ (*m*/*z* 820.5), [PC (36:3) + K]^+^ (*m*/*z* 822.5), [PC (38:6) + K]^+^ (*m*/*z* 844.5), and [PC (38:5) + K]^+^ (*m*/*z* 846.5), from tissue sections. Molecules were identified based on the fragmentation pattern caused by neutral losses. We assigned a molecule corresponding to [PC (32:0) + K]^+^ (*m*/*z* 772.5) as PC because we observed the neutral losses of 59 (*m*/*z* 713.5) and 183 (*m*/*z* 589.5), which correspond to a trimethylamine and a choline head group, respectively. The difference of 38 between *m*/*z* 551.5 and *m*/*z* 589.5 indicated that the adducted ion was changed from potassium to proton by fragmentation. We also observed the neutral loss of 256 from *m*/*z* 713.5 as *m*/*z* 457.2 in the MS/MS spectrum and speculated that it was palmitic acid (16:0). The MS Search of the Human Metabolome Database provides a candidate list of molecules corresponding to [PC (32:0) + K]^+^ (*m*/*z* 772.5), and taking into account the adducted ion and fragmentation pattern, we identified this molecule as [PC (16:0/16:0) + K]^+^ (Fig. 5a).

In the same manner, molecules corresponding to [PC (36:4) + K]^+^ (*m*/*z* 820.5), [PC (36:3) + K]^+^ (*m*/*z* 822.5), [PC (38:6) + K]^+^ (*m*/*z* 844.5), and [PC (38:5) + K]^+^ (*m*/*z* 846.5) were assigned as PC with potassium adduct because of the neutral loss of 59 or/and 183 in the MS/MS mass spectra. The molecules at [PC (36:4) + K]^+^ (*m*/*z* 820.5) and [PC (36:3) + K]^+^ (*m*/*z* 822.5) were identified as [PC (16:0/20:4) + K]^+^ (Fig. 5b) and [PC (16:0/20:3) + K]^+^ (Fig. 5c), respectively, because the neutral loss of 256 corresponds to palmitic acid (16:0). Based on the molecular weight, the other fatty acyl residues of these molecules were determined as arachidonic acid (AA, 20:4) and dihomo-γ-linolenic acid (DGLA, 20:3), respectively. The molecules at [PC (38:6) + K]^+^ (*m*/*z* 844.5) and [PC (38:5) + K]^+^ (*m*/*z* 846.5) were identified as [PC (18:2/20:4) + K]^+^ (Fig. 5d) and [PC (18:0/20:5) + K]^+^ (Fig. 5e), respectively, because neutral losses of 304 and 284 corresponding to AA (20:4) and stearic acid (18:0) were detected.

### Distribution of PC (16:0/16:0) in War-T

**Fig. 6:**
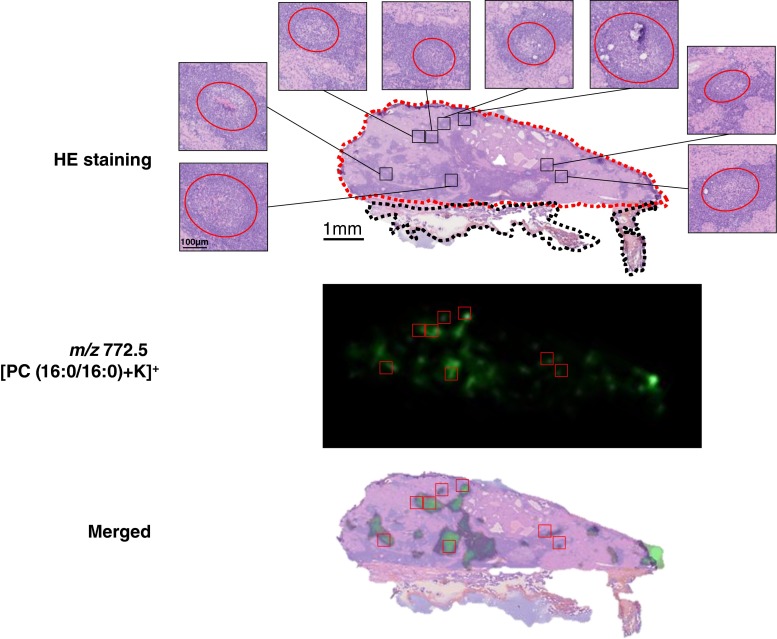
Location of PC (16:0/16:0) in the War-T region. The ion images of *m*/*z* 772.5, identified as [PC (16:0/16:0) + K]^+^, were merged with the HE staining image for case 1. According to the HE staining, we discriminated the neoplastic epithelium and lymphoid stroma on the basis of the description in Fig. 1. The ion image showed that the signal at [PC(16:0/16:0)+K]^+ (*m/z* 772.5)^ was more intense in the lymphoid stroma and performed clusters in the regions circled by *red lines*, folliculus lymphaticus

To compare the distribution of [PC (16:0/16:0)] and histological features, we merged the ion images and HE staining images (Fig. 6). By comparing the hot spots in the ion image and the enlarged HE staining images, we found that the strong signal of PC (16:0/16:0) was from the folliculus lymphaticus structure. This structure has a paler germinal center and an outer corona surrounded by numerous lymphocytes as we described in Fig. 1. From the merged images in Fig. 6, we observed that this signal was located mainly at the lymphoid stroma, especially accumulated in the folliculus lymphaticus.

## Discussion

War-T is remarkably soft compared to other tumors. This major feature is thought to be due to the formation of cysts and the included fluid. The PCs contained many long-chain, polyunsaturated fatty acids (PUFAs) as fatty acyl residues which could also be a reason for the softness of War-Ts, as such lipids provide fluidity of biological membranes [19, 20]. Regarding the fluidity of lipids, the transition temperature (*Tc*) is known as an important factor. The *Tc* was reported by Cullis et al. to be decreased in the lipids that contain fatty acyl residue with a *cis* double bond at C-9, and it is further lowered as the degree of unsaturation is increased [20]. In our study, most of the lipids detected in the War-T regions included two double bonds, such as PC (16:0/18:2). Some of them included fatty acyl residues with even four or five double bonds, such as PC (18:0/20:5) or PC (18:2/20:4). These unsaturated structures are thought to play a key role in the reduction of the *Tc* and to increase the fluidity of cell membranes in War-Ts, endowing War-Ts with their soft property.

In this study, we were able to pick up 15 peaks according to molecular species and the difference of signal intensities in case 1. Ten of the peaks showed significant differences in signal intensity (*p* < 0.01 in Table 2), and their specific localization at the War-T region or Non-T region were revealed (Fig. 3). Most of these signals have been studied in other tumor researches. PC (16:0/16:0), localized in the War-T region (Fig. 3), was detected in other types of tumor regions by Shimma et al. [21] and Chughtai et al. [22], but it was also found in normal regions by Ishikawa et al. [17]. On the other hand, the PCs located mainly in the War-T region, such as PC (16:0/20:4), PC (16:0/20:3) and PC (18:0/20:5) (Fig. 3), were reported as not significantly different between tumor and normal tissues in thyroid carcinoma study [17]. In addition, the signal *m*/*z* 796.5, significantly detected in the Non-T region in our present study (Fig. 3), was increased in the cancer region of thyroid carcinoma, and this PL was identified as PC (16:0/18:2) by MS/MS analysis [17]. Additionally, the signals of PC (16:0/16:0) and PC (16:0/18:2) showed no difference in distribution between cancer and stroma in oral squamous carcinoma [16]. The anatomic structures and the characteristic of these diseases might indicate different metabolisms in each tumor. Thus, IMS analyses enable us to easily detect the diversities of lipid composition which reflect different lipid metabolisms between tumor and non-tumor regions.

The War-T signals which had significantly different intensities (*p* < 0.01) according to the results of the statistical analysis were chosen for analysis in case 1 (Table 2, Fig. 3). From the ion images, all of these signals were observed mainly at the War-T region (Fig. 4). However, the signal at [PC(16:0/16:0)+K]^+ (*m/z* 772.5)^ showed a different localization compared to the other War-T signals. The merged ion images of [PC(16:0/16:0)+K]^+ (*m/z* 772.5)^ and [PC(36:2)+K]^+ (*m/z* 824.5)^ revealed a clear positional discrimination between these two biomolecules. By comparison with the HE staining images, the signal at [PC(36:2)+K]^+ (*m/z* 824.5)^ was found at neoplastic epithelium. In contrast, the signal at *m*/*z* 772.5 was located mainly in lymphoid stroma (Fig. 4). Especially, the folliculus lymphaticus in the War-T region exhibited high signal intensities at [PC(16:0/16:0)+K]^+ (*m/z* 772.5)^ (Fig. 6). These results suggest that compared to neoplastic epithelial cells, the lymphocytes have a different metabolism pathway in War-T.

Stearoyl-CoA desaturase-1 (SCD1) is a microsomal enzyme that regulates the conversion of saturated fatty acids (SFAs) such as palmitic acid (C16:0) and stearic acid (C18:0) into mono-unsaturated fatty acids (MUFAs) such as palmitoleic (C16:1) and oleic acid (18:1) [23]. The production of MUFAs from SFAs is thus thought to be impeded by the absence of SCD1, leading to the decrease of PCs which are incorporated by MUFAs or PUFAs. Ide et al. observed increased SCD1 in breast cancerous areas, where PCs including MUFAs such as PC (32:1), PC (34:1), and PC (36:1) were detected predominantly. In contrast, the PCs with SFAs such as PC (32:0), PC (34:0), and PC (36:0) were decreased in the cancer [15].

In our present study, the signal at *m*/*z* 772.5 identified as [PC (16:0/16:0) + K]^+^ (Fig. 5) also includes only SFAs as fatty acyl residues, and it was found mainly in the lymphoid stroma, especially folliculus lymphaticus, and not in the neoplastic epithelium. This result suggests that a deficiency of SCD1 activity might lead to the accumulation of PC (16:0/16:0) in folliculus lymphaticus of War-T. Buttke et al. reported a deficiency of SCD activity in murine T lymphocytes [24]. SCD is known to have a wide variety of isoforms. In a study by Tebbey et al., neither B lymphocytes nor T lymphocytes in mice expressed SCD1 [25]. Although there is little information available about SCD1 in human lymphocytes, a deficiency of SCD1 might be one of the results of the accumulation of PC (16:0/16:0) in the folliculus lymphaticus of War-Ts.

In this study, we also focused on the fatty acyl residues bound to PC species in the War-T regions, because the fatty acyl residue is expected to reflect some features of War-T, such as metabolism during the pathological process. A study of War-T by Andreadis et al. showed that ICAM-1, a cell adhesion factor, was strongly expressed in the germinal center of folliculus lymphaticus and moderately expressed in many epithelial cells and more specifically on the whole membrane of basaloid cells and the luminal surface of columnar cells [8]. ICAM-1 is involved in the interaction between lymphocytes and epithelial cells [26]. Palmitic acid (16:0) was reported to upregulate the expression of ICAM-1 in epithelial cells [27]. Therefore, palmitic acid might participate in the interaction between neoplastic epithelial cells and lymphocytes by the regulation of ICAM-1 expression. In fact, our present findings demonstrated that PC (16:0/16:0), which is containing only C16:0 as fatty acyl residues and can release only palmitic acid by phospholipase, was increased in the lymphoid stroma of the War-T regions, especially in the folliculus lymphaticus.

Some studies suggested that an immunological reaction is involved in the formation of War-T, which should be considered a delayed hypersensitivity disease with persistent inflammatory reactions [4, 6, 28], and the observation of an infiltration of lymphoid cells and a number of immunohistochemical findings also supported the importance of immune activation in the pathogenesis of War-T [29]. Palmitic acid was recently reported to induce an upregulation of inflammatory factors such as interleukin-6 and tumor necrosis factor-α [30]. In our study, PC (16:0/16:0) was increased in the lymphoid stroma, especially the folliculus lymphaticus, in the War-T region. This accumulation might be involved in the inflammatory reaction in War-T.

In this study, we focused on the analysis and discussion of PC (16:0/16:0) specifically distributed in the folliculus lymphaticus of lymphoid stroma in War-T; however, there are other PC species which have distinct distributions in War-T as indicated in the figure. Especially, PC species (16:0/20:4), (18:2/20:4), and (16:0/20:5) were significantly increased in both neoplastic epithelium and lymphoid stroma. PC (16:0/20:3) was specifically located in neoplastic epithelium. These PCs contained long-chain polyunsaturated fatty acyl residues. Long-chain polyunsaturated fatty acids (LC-PUFAs) as fatty acyl residues present in PCs have been described to be precursors of lipid inflammatory mediators after their release by the activation of phospholipases. The LC-PUFAs could be involved in inflammatory responses [31], such as AA (20:4), one kind of *n*-6 series fatty acid, which was reported to participate in inflammatory reaction [32], and the elevated level of AA-contained PCs were considered related to chronic inflammation in other studies [33, 34]. In our study, PC (16:0/20:4) and PC (18:2/20:4) containing AA were increased in War-T region. In addition, DGLA, (C20:3), which is the immediate precursor of AA, and linoleic acid (LA, C18:2), which is the essential FA for human and the precursor of *n*-6 series fatty acids, were discovered combining in PC (16:0/20:3) and PC (18:2/20:4) in War-T. Therefore, these PC species containing LC-PUFAs might be related to the inflammatory response and development of War-T and are potentially markers for War-T lipid fingerprinting.

## Conclusion

An IMS analysis revealed that 10 signals have significant differences in signal intensities between the War-T and Non-T regions from case 1. There were nine signals specifically detected in the War-T region. After having applied these War-T positive signals to all dataset, five War-T specific signals were confirmed: one signal, identified as PC (16:0/20:3), was located mainly in neoplastic epithelium; three signals identified as PC (16:0/20:4), PC (18:2/20:4), and PC (18:0/20:5), were located mainly in neoplastic epithelium and lymphoid stroma; and one signal, identified as PC (16:0/16:0), was specifically distributed in the lymphoid stroma, especially folliculus lymphaticus in War-T.
